# Combined Tumor Cell and Lysate-Based Vaccines for Immunotherapy of Primary and Recurrent Glioblastoma (GBM)

**DOI:** 10.3390/cancers17233772

**Published:** 2025-11-26

**Authors:** Apostolos Stathopoulos, Philippe Glorieux, Evangelos M. Rokas, Huub F. J. Savelkoul

**Affiliations:** 1 Epitopoietic Research Corporation, 5032 Gembloux Isnes, Belgium; tstath@hotmail.com; 2 Department of Neurological Surgery, Keck School of Medicine, University of Southern California, Los Angeles, CA 90033, USA; 3 Cell Biology and Immunology Group, Wageningen University and Research, 6700 AH Wageningen, The Netherlands; 4 Hematology-Oncology Department, Cliniques du Sud-Luxembourg, Vivalia, 6700 Arlon, Belgium; philippe.glorieux@vivalia.be; 5 Neurosurgery Department, Athens Medical Center, 151 25 Athens, Greece; evrokas@gmail.com

**Keywords:** recurrent glioblastoma, cancer transplant immune recognition therapy (CTIRT), GBM vaccine, immunotherapy, allogeneic CTL

## Abstract

Glioblastoma (GBM) remains the most aggressive primary brain tumor, characterized by extensive intra-tumoral heterogeneity, profound local immunosuppression, and a highly adaptive tumor microenvironment that resists conventional therapies. Immunotherapy for GBM tries to overcome these barriers by reactivating anti-tumor immunity through cellular, molecular, and immune-modulatory interventions. The therapeutic efficacy of the cell-based vaccines in patients with glioma and glioblastoma is primarily driven by tumor antigen-specific CD8^+^ T cell activation, orchestrated by CD4^+^ T cell help. Several whole-cell vaccine platforms (e.g., DCVax-L, CMV-targeted vaccines, and Cancer Transplant Immune Recognition Therapy (CTITR)) provide personalized formulations. CTITR consists of irradiated autologous and allogeneic glioma cells and their lysates, leveraging the inherent immunogenicity of allogeneic material to bypass the need for predefined tumor-specific antigen selection. This strategy promotes broad CD8^+^ T cell expansion, potentially exceeding 10^9^ antigen-specific cytotoxic T lymphocytes, sufficient for substantial tumor clearance. Such a preparation can start with approximately 1 g of surgically resected tumor tissue per patient to generate both autologous and allogeneic vaccine components. Clinical observations indicate that cell-based vaccine preparations can be effective in both newly diagnosed glioblastoma patients treated post-surgery and in patients with recurrent gliomas. Cell-based vaccines, including CTITR, offer novel, antigen-agnostic immunotherapeutic platforms that harness autologous DC and autologous and allogeneic glioma cells, and their lysates bypass the need for predefined tumor-specific antigen selection.

## Simple Summary

Glioblastoma (GBM) carries a poor prognosis, and new therapeutic strategies are necessary. Besides peptide- and tumor-lysate-loaded DC vaccination, Cancer Transplant Immune Recognition Therapy (CTIRT) is based on vaccines using isolated tumor cells. CTIRT uses cells and their lysates of the resected tumor cells of the patient and those of other individuals. The potency of these cell-based vaccines is based on the generic activity of allogeneic cells to promote the activation of CD8^+^ T-cells, without the need to identify and employ tumor-specific antigens. In addition, this ensures the expansion of the pool of CD8^+^ T cells to a population of more than 10^9^ CD8^+^ T cells, which can reduce the existing tumor load. Such vaccines can be effective in halting glioblastoma, both in primary diagnosed patients who undergo surgery and receive the treatment, but also in patients with recurrent gliomas who receive such vaccine treatments.

## 1. Introduction

### 1.1. Glioblastoma and Gliomas

Gliomas represent over 26% of all primary brain tumors, with glioblastoma (GBM) being the most prevalent and aggressive malignant subtype in adults. GBM accounts for approximately 14% of all brain tumors, 51% of malignant brain tumors, and nearly half of all primary malignant central nervous system (CNS) neoplasms. Despite aggressive multimodal treatment—including surgical resection, radiotherapy, and chemotherapy—median overall survival remains between 12 and 18 months, and 5-year survival rates are below 7% [1].

This dismal prognosis is driven by the intrinsic resistance of glioblastoma cells to standard therapies, supported by robust DNA repair mechanisms and the presence of treatment-resistant tumor-initiating cells [2]. It was shown that GBM stem cells display a modified epigenetic landscape by the production of phosphocreatine (PCr), which promotes accurate chromosome segregation and tumor cell proliferation. Pharmacological disruption of PCr biosynthesis blocks tumor growth [3]. The molecular heterogeneity of GBM, characterized by the absence of uniform antigenic targets, further complicates treatment development.

### 1.2. Standard and Recurrent GBM Management

First-line treatment for newly diagnosed GBM consists of maximal safe surgical resection, followed by concurrent radiotherapy and temozolomide chemotherapy, with adjuvant temozolomide continuation [4]. Despite this intensive regimen, recurrence is almost universal (>95%) within 9–12 months. The 5-year survival remains around 5%, and median survival after recurrence is less than one year. Less than half of patients with recurrent GBM are eligible for repeat surgical intervention, and no standardized second-line therapy currently exists [5,6]. This clinical landscape underscores the urgent need for novel therapeutic strategies capable of circumventing the limitations of conventional approaches [7].

Immunotherapy refers to therapies that harness the immune system to target tumors and exploit the presence of tumors of rather unique tumor-specific antigens that are able to bind to T-cell receptors or antibodies. The main categories of immunotherapy protocols comprise immune checkpoint inhibitors, tumor vaccines, and adoptive cell therapy (ACT). The purpose of these therapeutic protocols is to restore the weakened immune system and thereby, restoration of residual or disseminated tumor cells [8]. Promising application potential of immunotherapy was shown in the management of various malignancies by enhanced therapeutic effect, prolonged patient survival, and improved patient quality of life. However, immunotherapy resistance in glioblastoma has resulted in limited clinical success, although increasing insights continue to make immunotherapy an effective option in GBM treatment [9].

### 1.3. The Immunosuppressive GBM Environment

The success of these immunotherapies relies on the priming of tumor-specific T cells within the tumor microenvironment (TME) [10]. However, the heterogeneous nature of the tumor but also the TME and the various immune evasion mechanisms of the tumor provide a major challenge to the design, efficacy and safety of immunotherapy approaches. Chemotherapeutic agents and corticosteroids exacerbate immune dysfunction by inducing lymphopenia, depleting CD4^+^ T cells, and expanding regulatory T cell (Treg) populations. Nevertheless, the identification of neoantigens, tumor-specific mutations not present in normal tissue, provides a promising avenue for immunotherapy. However, the role of tumor mutational burden or mismatch-repair deficiency are largely unknown in GBM, although these factors were suggested to be a predictor for the response to treatment with immune checkpoint inhibitors [11,12]. These checkpoint inhibitors are monoclonal antibodies that block inhibitory signaling pathways that regulate T-cell activation and include CTLA-4 (cytotoxic T-lymphocyte-associated protein 4) and PD-1 (programmed cell death protein 1). Tumors often upregulate ligands for these receptors on T-cells, including PD-L1, which suppresses T-cell cytotoxic activity [13,14]. When these signals are blocked, e.g., by ipilimumab targeting CTLA-4, or nivolumab and pembrolizumab targeting PD-1, T-cells remain active, proliferate, secrete cytokines and induce lysis of tumor cells [15]. However, their efficacy depends on the tumor mutational burden and the tumor immune microenvironment [16,17,18].

A recent report described a treatment-naïve GBM patient receiving immune checkpoint inhibitors (anti-PD-1, anti-CTLA-4, anti-LAG3) prior to surgical resection, resulting in prolonged disease-free survival and enhanced tumor-infiltrating lymphocyte activation [19]. Immune cells in the TME exhibit dual functionality: while initially capable of eliminating malignant cells (cancer immunosurveillance), they can also promote tumor growth (cancer immunoediting). In this dynamic interplay, immune pressure selects for less immunogenic tumor clones, while immune cell function is progressively diminished by the TME’s suppressive cues. Overcoming these barriers is critical for the success of immunotherapeutic strategies in GBM.

### 1.4. Immunotherapeutic Strategies

Cancer immunotherapy aims to harness the host’s immune system to recognize and eliminate malignant cells [20]. The current immunotherapeutic strategies for glioblastoma comprise vaccine-based immunotherapies with cell-based and peptide vaccines to induce tumor-specific adaptive immunity by exposing the immune system to tumor antigens [21,22]. Two candidate vaccines that show clinical efficacy are dendritic cell (DC) vaccines, such as DCVax-L [23], and Sitoiganap (ERC1671/Gliovac^®^) [24,25]. In addition, peptide vaccines, such as Rindopepimut (EGFRvIII peptide conjugated to KLH), were used but did not show clinical effects [26]. Also, heat-shock protein–peptide complex vaccines (e.g., HSPPC-96, Prophage G-200) can deliver autologous chaperone-bound tumor peptides with the goal to stimulate DC and T-cell priming [27]. Apart from cell-based vaccines, immune checkpoint inhibitors that target PD-1/PD-L1 (nivolumab, pembrolizumab) and CTLA-4 (ipilimumab) are also increasingly being used, but clinical efficacy is limited due to the immunosuppressive microenvironment and the low mutational burden in GBM [28,29]. Next, selective oncolytic viruses are used as they can infect and lyse tumor cells while releasing neoantigens and danger-associated molecular patterns (DAMPs) that promote systemic anti-tumor immunity [30]. These viruses comprise DNX-2401 (Delta-24-RGD adenovirus), HSV G207, and PVSRIPO (polio–rhinovirus chimera), each demonstrating safety and durable responses in subsets of patients [31,32]. When combined with immune checkpoint inhibitors, the therapies might potentiate immune priming.

Increasingly, attention is generated for the applications in GBM of adoptive cell therapies (ACTs) such as chimeric antigen receptor (CAR) and receptor-engineered T cells, tumor-infiltrating lymphocytes (TILs), and NK-cell-based approaches that target specific tumor antigens. Although clinical efficacy remains limited, the current generation of multiantigen CARs can potentially overcome tumor antigen escape and immune suppression [33].

Since the TME is dominated by tumor-associated macrophages (called microglia) and regulatory T cells, cytokine-based therapies targeting CSF1R, TGF-β, IDO1, and VEGF signaling are under evaluation for their contribution to clinical efficacy [34]. The VEGF-A inhibiting monoclonal antibody, bevacizumab, is now part of different GBM treatment schedules [35]. Recently, experimental applications of neoantigen prediction and RNA sequencing technologies have been used to determine the tumor’s mutations and thereby allowing the tailoring of necessary T-cell responses to the individual GBM patient [15,36,37].

Glioblastoma is notoriously resistant to T cell-mediated immunotherapy due to a combination of immune checkpoint expression, lack of sufficient tumor-infiltrating lymphocytes (TILs), and a highly suppressive tumor microenvironment (TME). In addition, the infiltration of T-cells into the TME, along with extensive myeloid infiltration and disruption of the blood–brain barrier (BBB), significantly impede immune-mediated tumor clearance [38,39]. Ideally, anti-tumor therapy should induce a strong correlation between T-lymphocyte counts and overall survival in GBM [40,41,42]. However, the overall prognosis for GBM remains poor, with many patients still facing limited treatment options and high recurrence rates [42].

Recently, the gut microbiota is implicated in glioma development, progression and responsiveness to treatment, as these can impact the tumor microenvironment, although the underlying mechanisms remain largely unknown [43]. Intestinal microbiota affects the development of the immune system, but also modulates innate and adaptive immune responses and induces the secretion of pro-inflammatory factors, thereby influencing the TME and subsequent efficacy of the applied anti-tumor immunotherapy protocols [44,45]. As a result, microbial components may aid in the overall effectiveness of immunotherapy.

### 1.5. Cell-Based GBM Immunotherapy

Immune cell therapy intends to boost anti-cancer immune responses by supplying cells with the desired specificity and functionality to the patient [46]. However, of a collection of 230 cancer patients, about 230 neoantigens were identified, of which only 11% induced specific T cell responses, demonstrating the difficulty of identifying optimal antigens for personalized vaccines [47]. GBM vaccines are generally developed from freshly obtained tumors from the individual patient, ensuring a personalized treatment and using the complete set of relevant tumor-associated antigens [48,49,50]. DCs can either be therapeutically targeted in vivo in the patient or ‘‘educated” ex vivo by isolating their blood-derived monocyte precursors from a cancer patient, and after manipulating them ex vivo, injecting them back into the same patient [51]. Because of their extensive expression of PRR, these DC are able to recognize tumor-derived PAMPs and show enhanced presentation of these antigens to tumor-specific T-cells, thereby decreasing the expression of tolerogenic markers PD-L1 [52,53]. To further support T cell priming, dendritic cells can be expanded and activated ex vivo using cytokines such as the granulocyte–macrophage colony-stimulating factor (GM-CSF), which bypasses the immunosuppressive signaling present in the tumor microenvironment, enables controlled activation and maturation of DCs, and ensures the delivery of tumor antigens in an immunogenic context [54,55]. The result is an efficient and personalized immune education process that culminates in the generation of high-affinity CTLs with the potential to mediate tumor regression [56].

**Figure 1:**
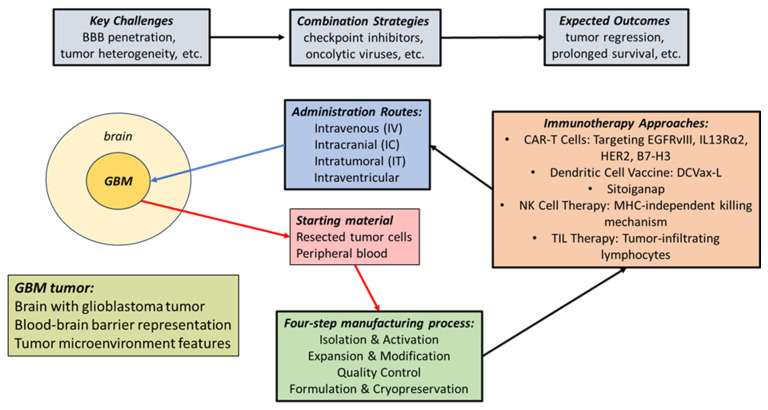
The approaches for the design and production of cell-based immunotherapy preparations for GBM.

Personalized vaccines based on autologous tumor lysates are tailored to unique patient-related tumor antigens. Lysates of these tumor cells allow exposure to all tumor-associated antigens (see Figure 1 for a schematic outline of the widely published procedures for the design and production of cell-based immunotherapy preparations for GBM) [57]. Subsequent presentation of these tumor-specific antigens by dendritic cells in vivo ensures the induction of a robust immune response [58,59]. In addition, in vitro antigen loading by tumor lysates permits DC to activate cytotoxic T-cells upon infusion into the patient and shows functional potency by improving subsequent T-cell vaccination responses [58,60]. Primed DC enhances T-cell activation by also utilizing cytokines, like IL-6 and TGF-beta, which are produced by GBM, to promote Th17 cell generation [60,61]. In addition, this enhanced immune recognition of tumor antigens, together with the secretion of pro-inflammatory cytokines, facilitates the interaction with native immune cells, potentially initiating a self-propagating cancer-immunity cycle that converts the immunosuppressive tumor microenvironment into one that supports anti-tumor immunity [62,63].

Several trials have demonstrated that patients receiving such personalized vaccines exhibit improved progression-free survival (PFS) and overall survival (OS). For instance, median OS was reported at 16 months, with a notable percentage of patients remaining alive at 3 years [60]. Subsequently, a significant proportion of patients (90%) showed immune responses to the personalized peptides, correlating with prolonged survival, particularly in those with multiple vaccine-induced responses [64]. Generally, cell-based vaccines are well-tolerated, with most adverse events being mild, such as local injection site reactions [60,65]. This safety profile supports the feasibility of integrating personalized vaccines into standard treatment regimens. While personalized vaccines show promise in enhancing immune specificity and improving clinical outcomes in GBM patients, challenges remain in standardizing treatment protocols and ensuring widespread accessibility. Further research is needed to optimize these therapies and fully understand their long-term effects.

## 2. Therapeutic GBM Tumor Vaccines

### 2.1. Anti-Tumor T Cell Responses

Immunotherapy remains a challenging approach due to the heterogeneous nature of the tumor and the TME and the extensive immune evasion mechanisms [66]. Among the most potent effectors in this response are antigen-specific CD8^+^ cytotoxic T lymphocytes (CTLs), which can directly lyse tumor cells upon recognition of peptides presented on MHC class I molecules on dendritic cells (DCs) and receiving help through cytokine-mediated signaling networks from CD4^+^ helper T cells [67]. The hallmark of tumor vaccines is to prime rare, tumor-specific CD8^+^ T cells from the naïve repertoire and expanding them into large effector pools capable of mediating cytotoxic activity, e.g., an in vitro ≥1000-fold expansion in two months [68,69,70]. In vivo expansion may be more modest (10- to 25-fold), but cross-reactivity within the T cell receptor (TCR) repertoire can enhance specificity and functional diversity [71]. Dendritic cells serve as central coordinators in the immune cascade by capturing tumor-derived antigens, migrating to lymphoid tissues, and presenting these antigens to naïve T cells. CD4^+^ T cells of the Th1 subset are essential for effective anti-tumor immunity by their secretion of cytokines such as IFN-γ, which enhance DC function and T cell cytotoxicity, and support the licensing of DCs through CD40–CD40L interactions [72]. These DCs not only activate T cells but also recruit them to the TME, where the T cells interact with the tumor and kill them by the release of cytotoxic effector molecules like interferon-γ (IFN-γ), granzyme B and perforin [73]. However, the minor response of GBM to immunotherapy appears to be due to the limited number of cytotoxic T cells infiltrating the TME, while there is substantial myeloid cell infiltration and a substantial alteration of the blood–brain barrier [74]. Furthermore, CD4^+^ T cells augment the survival and functional maturation of CD8^+^ CTLs and can even exert direct cytotoxicity under certain conditions [75].

Although CD8^+^ T cell infiltration has been observed in GBM tissues, the tumor typically evades immune detection through multiple mechanisms, including downregulation of MHC expression and induction of systemic immunosuppression. The immunosuppressive microenvironment is largely based on the presence of tumor-associated macrophages, mostly of the M2 phenotype and characterized by the secretion of anti-inflammatory cytokines IL-10 and TGF-β that inhibit the activity of Th1-polarized CD4^+^ and cytotoxic CD8^+^ T cells to initiate a cytolytic anti-tumor response while inducing the recruitment of regulatory Treg cells due to the release of chemokines like CCL2 and CCL22 [76]. This leads to further suppression of the CD8^+^ Tc effector cells and decreased killing of tumor cells. In addition, myeloid-derived suppressor cells releasing reactive oxygen species (ROS), nitric oxide (NO) and immunosuppressive cytokines further inhibit the activation and function of tumor-specific T cells [77]. Depletion of regulatory T cells (Tregs), for instance, via low-dose cyclophosphamide, has been shown to improve anti-tumor responses in preclinical GBM models by reducing IL-10 production and enhancing effector T cell function [78,79]. In addition, strategies that enhance the antigen load presented to DCs, such as mixing autologous and allogeneic tumor-derived antigens, can improve the breadth and magnitude of T cell responses [80]. By designing and using vaccines by introducing tumor lysates from both self- and HLA-mismatched donors, facilitating both self-specific and alloreactive immune activation thereby leverages this principle [24,41,81,82]. Nevertheless, the overall prognosis for GBM remains poor, with many patients still facing limited treatment options and high recurrence rates [83].

Moreover, the expansion and persistence of CD8^+^ T cells are regulated by cytokines, including IL-7, which supports naïve CD8^+^ T cell survival; IL-15, which promotes memory T cell proliferation; and IL-21, which synergizes with IL-15 to enhance IFN-γ production and CTL activation [84]. In contrast, IL-2 may inadvertently expand immunosuppressive Tregs, dampening anti-tumor responses. In solid tumors, T cells face formidable barriers imposed by the immunosuppressive tumor microenvironment (TME), including loss of MHC expression, recruitment of tolerogenic APCs, secretion of inhibitory cytokines (e.g., TGF-β, IL-10), and upregulation of immune checkpoint ligands such as PD-L1 [85].

While tumor-specific T cell precursors are scarce (typically 0.002–0.4%), alloreactive T cells, which recognize non-self MHC molecules, exist at significantly higher frequencies (ranging from 0.7% to over 20%) [86,87,88]. This disparity presents an opportunity: by introducing allogeneic tumor cells expressing non-self MHC, one can harness the abundance of alloreactive CD8^+^ T cells to drive a potent anti-tumor response. Given the diversity of the human TCR repertoire (∼10^8^ unique clonotypes in a healthy adult), cross-reactivity can further broaden the immune response, increasing the chances of recognizing diverse tumor antigens [89]. Following vaccination, in vivo expansion of tumor-reactive CD8^+^ T cells can reach up to 10^9^–10^10^, which is considered the threshold for effective tumor debulking [90,91]. Cell-based vaccines employing multi-donor allogeneic tumor cells to amplify the repertoire of presented neoantigens enables the generation of a polyclonal CTL response targeting multiple epitopes, enhancing the likelihood of tumor clearance and minimizing immune escape [92]. By combining autologous and allogeneic tumor cells and lysates, such a vaccine not only stimulates endogenous tumor-specific CTLs but also induces alloreactivity, resulting in the recruitment and activation of a broader range of effector T cells [93,94]. By injecting a vaccine containing allogeneic tumor cells and lysate derived thereof, pro-inflammatory allogeneic dendritic cells are properly activated, break the immunosuppressive effect of the TME, and efficiently prime specific anti-tumor T-cell responses [95]. The resulting IL-2 levels are sufficient to expand tumor-specific T cells without inducing the generation of immunosuppressive Treg cells. This dual mechanism supports therapeutic efficacy, even in patients where the tumor is unresectable or resistant to prior treatments. The magnitude of CTL activation can be determined via IFN-γ production [96], and the presence of tumor-specific cytotoxic responses has been confirmed in preclinical and clinical settings.

### 2.2. Responding Antigen-Presenting Cells

The initiation of effective cytotoxic T lymphocyte (CTL) responses requires robust antigen presentation by professional antigen-presenting cells (APCs), particularly dendritic cells (DCs) [97]. Upon tumor cell death, induced in vivo by therapy or immunologic attack or ex vivo by lysing resected tumor cells, tumor antigens are released into the microenvironment, where they are captured and processed by APCs such as macrophages and DCs [98]. During this process, dying tumor cells release damage-associated molecular patterns (DAMPs), including high-mobility group box 1 (HMGB1) and heat-shock proteins (e.g., HSP70), which serve as endogenous danger signals. These signals activate DCs through pattern recognition receptors (PRRs, including toll-like receptors or TLRs), leading to DC maturation and the cross-presentation of tumor-associated antigens (TAAs) on MHC class I and II molecules [99,100]. This also leads to upregulation of costimulatory molecules (CD80/CD86) and cytokine production (e.g., IL-12, TNF-α), thereby promoting both CD8^+^ cytotoxic T-cell and CD4^+^ helper T-cell responses [101]. Such immunogenic lysates can therefore serve a dual function: providing a source of antigens representing the tumor’s full mutational and neo-antigenic repertoire and displaying intrinsic adjuvant activity by triggering innate immune activation without the need for exogenous adjuvants. This principle underlies the clinical strategies of cell-based vaccines. By using different defined TLR ligands (or complete whole tumor cell lysates) in combination with intact tumor cells, DC can be actively stimulated and inhibit tumor growth in preclinical GBM tumor models [102]. Such enhanced T-cell priming while concomitantly suppressing immune inhibitory signals due to interaction with costimulatory signals can convert immunologically cold tumors to hot tumors promoting immune-based clearance of the tumor. Such therapeutic platforms can simultaneously overcome GBM tumor heterogeneity and therapeutic resistance [103,104,105].

Activated DCs express high levels of co-stimulatory molecules (CD40, CD80, CD86) and secrete cytokines such as IL-12, type I interferons (IFNs), and IL-15, which are essential for priming CD8^+^ and CD4^+^ T cells. This interaction not only induces cytotoxic differentiation of CD8^+^ T cells but also generates helper CD4^+^ T cells that further support CTL survival and function. Notably, IL-15 enhances CTL proliferation and memory formation, while excessive IL-2 levels may promote regulatory T cell (Treg) expansion and suppress anti-tumor immunity [95,106,107]. Importantly, CD4^+^ T cells play a critical role in licensing DCs via CD40-CD40L interactions, enabling optimal activation of CD8^+^ T cells. CD4^+^ T cells can also exert direct cytotoxic effects on MHC class II-expressing tumor cells. However, this function may be counterbalanced by the presence of Tregs, which suppress immune responses and contribute to tumor immune evasion. Injection of irradiated allogeneic tumor cells and lysates generates a local pro-inflammatory response driven by MHC mismatch, which in turn activates host DCs within the tumor microenvironment. This process bypasses the need for ex vivo loading of DCs, as the immune activation occurs in situ via recruitment, maturation, and antigen loading of endogenous DCs. This in vivo licensing mechanism facilitates both MHC class I-restricted presentation to CD8^+^ T cells and class II-restricted presentation to CD4^+^ T cells, driving a coordinated and polyclonal anti-tumor immune response. Critically, the level of IL-2 induced is sufficient to expand tumor-reactive T cells while remaining below the threshold required to expand Tregs, thus preserving immune effector function.

To further support T cell priming, dendritic cells can be expanded and activated ex vivo using cytokines such as granulocyte–macrophage colony-stimulating factor (GM-CSF). DCs exposed to tumor lysates derived from both autologous and allogeneic sources ensure presentation of a broad range of tumor epitopes upon reinfusion. This strategy provides several advantages: it bypasses the immunosuppressive signaling present in the tumor microenvironment, enables controlled activation and maturation of DCs, and ensures the delivery of tumor antigens in an immunogenic context. The result is an efficient and personalized immune education process that culminates in the generation of high-affinity CTLs with the potential to mediate tumor regression. Granulocyte-Colony Stimulating Factor (G-CSF)-mobilized, HLA-mismatched donor tumor cells have been shown to enhance CD8^+^ T cell frequencies, with therapeutic efficacy correlating with the degree of expansion [108]. In vivo, IFN-γ produced by activated T cells enhances antigen presentation, upregulates MHC expression, and promotes tumoricidal macrophage differentiation [109]. Classically activated (M1-like) macrophages possess antigen-independent tumoricidal properties and can enhance T cell priming. While myeloid-derived suppressor cells (MDSCs) and tumor-associated macrophages (TAMs) often inhibit anti-tumor immunity, certain conditions—such as IFN-γ exposure—can reprogram these populations toward a pro-inflammatory, anti-tumor phenotype. However, the glioma microenvironment can also shift myeloid cells toward an immunosuppressive M2-like state. IL-33, a glioma-derived alarmin, plays a pivotal role in this phenotypic switch [110]. When IL-33 is sequestered in the nucleus of tumor cells, it drives reprogramming of TAMs toward tumor-supportive functions, facilitating immune evasion and rapid tumor progression.

### 2.3. Tumor-Suppressive Environment (TME)

GBM creates a highly immunosuppressive microenvironment that inhibits effective immune responses, including T-cell dysfunction and the presence of regulatory T cells [111,112]. Also, various immunosuppressive cytokines, such as TGF-β and IL-10, are present [113]. The presence of tumor-associated macrophages (TAMs) and myeloid-derived suppressor cells (MDSCs) further contributes to immune evasion [114,115]. The glioblastoma (GBM) tumor microenvironment (TME) is characterized by a complex immunosuppressive network composed of tumor-infiltrating immune cells, stromal elements, and soluble mediators. Myeloid-derived populations, such as tumor-associated macrophages (TAMs), microglia, neutrophils, and myeloid-derived suppressor cells (MDSCs), constitute the majority of immune cells within the GBM TME, while lymphocytes account for only 10–15% of the infiltrating leukocytes. This immunosuppressive milieu promotes glioma growth, immune evasion, and resistance to therapy [116]. TAMs, especially those polarized to an M2-like phenotype, secrete anti-inflammatory cytokines (e.g., IL-10, TGF-β), promote angiogenesis, remodel the extracellular matrix, and suppress cytotoxic T lymphocyte (CTL) activity. These effects are compounded by the secretion of chemokines (e.g., CCL2, CCL22), which recruit regulatory T cells (Tregs) and inhibit effector T cell infiltration and function.

Due to the rapid proliferation of tumor cells and the disorganized vasculature, the microenvironment of the tumor is hypoxic and nutrient-deprived, thereby modulating CD8^+^ T cell development and effector functions while tumor growth and metastasis induction are promoted [117]. Moreover, glycolysis is increased due to the metabolic shift as hypoxia induces HIF-1α (hypoxia-inducible factor-1α), which transcriptionally upregulates key glycolytic enzymes and glucose transporters (e.g., GLUT1) while repressing mitochondrial respiration genes. This promotes glycolysis by converting glucose to lactate rather than relying on mitochondrial oxidative phosphorylation (OXPHOS) even under limited oxygen availability (the Warburg effect) [118,119]. Hypoxic conditions also upregulate the expression of immune checkpoint molecules on T cells and inhibit CTL proliferation and effector functions, further favoring immune escape.

The tumor’s ability to evade immune detection is exacerbated by its heterogeneity, which complicates the targeting of specific tumor antigens [9,120]. Strategies that disrupt the immunosuppressive roles of myeloid cells in the tumor microenvironment can improve the efficacy of tumor lysate-based therapies [58]. Combining tumor lysates with immune checkpoint inhibitors can further enhance T cell activation and proliferation against GBM [121]. While these mechanisms show promise, challenges remain in overcoming the immunosuppressive environment of GBM, which may limit the effectiveness of these therapies. Ongoing research is essential to refine these strategies and improve patient outcomes.

### 2.4. Resistance Mechanisms

Resistance to immunotherapy is prevalent, with various mechanisms such as tumor plasticity and intra-tumoral heterogeneity contributing to treatment failure [9,122]. The lack of immunogenicity and universal identifying features in GBM cells diminishes the efficacy of therapies like immune checkpoint inhibitors and CAR T-cell therapies [111,123]. The use of tumor cells and lysates in immunotherapy for Glioblastoma Multiforme (GBM) can stimulate an immune response through several mechanisms. These approaches leverage the unique properties of tumor antigens and the immune system’s ability to recognize and attack cancer cells. Tumor lysates can be used to pulse dendritic cells, enhancing their ability to present tumor-specific antigens to T cells, thereby initiating an immune response [124]. Autologous tumor lysates can be tailored as personalized vaccines to individual patients, increasing the likelihood of a robust immune response against specific tumor antigens [125,126,127]. In addition, immune modulation can be used by the integration of tumor lysates with agents like levamisole that can stimulate immune activity as combination therapy, thereby counteracting the immunosuppressive tumor microenvironment typical of GBM [125]. The recent developments enabling the use of CAR-T cells targeting specific antigens, such as IL13Rα2, in conjunction with tumor lysates can enhance the effectiveness of the immune response by promoting direct interactions between modified T cells and tumor cells [125]. Despite these challenges, ongoing research aims to develop combination therapies and novel strategies to enhance the efficacy of immunotherapy in GBM. However, the complexity of the tumor and its environment continue to pose significant hurdles that require innovative solutions [21,22].

Inflammation contributes to protective anti-tumor immune responses while maintaining tissue homeostasis. However, during GBM cancer development, this inflammatory response is non-resolving, and this might be caused by cancer-initiating mutations, thereby contributing to tumor progression [128,129]. Thus, the chronic, non-resolving inflammation contributes to tumor progression, rather than suppression. This inflammation is initiated by tumor-derived signals, including IL-1, IL-6, and IL-33, which activate surrounding stromal and immune cells. Over time, this leads to the recruitment of immunosuppressive populations (e.g., M2 macrophages, Tregs, MDSCs), further reinforcing immune evasion and tumor proliferation [110]. Moreover, chronic inflammation in GBM is closely linked to epigenetic changes, such as DNA methylation, histone modification, and noncoding RNA regulation [129]. These alterations affect inflammatory gene expression, support immune suppression, and contribute to oncogenic transformation and therapy resistance.

Treatment regimens of GBM include the use of temozolomide (TMZ), the working mechanism of which is primarily based on DNA alkylation, leading to cytotoxic DNA damage and apoptosis of tumor cells [21,22,130,131]. In addition, TMZ activity is also linked to the epigenetic activation of DNA repair mechanisms [15,132].

### 2.5. Tumor-Specific Peptide Sequences in Glioblastoma

Peptide-based cancer vaccines utilize immunogenic epitopes derived from tumor-associated antigens (TAAs) or tumor-specific neoantigens to elicit targeted T cell responses [133,134]. These epitopes are typically presented on the surface of tumor cells via MHC class I (for CD8^+^ T cells) or class II (for CD4^+^ T cells) molecules [135]. It is known that high-avidity CD8^+^ cytotoxic T lymphocytes (CTLs) targeting self-derived TAAs are rare in the naïve T cell repertoire due to thymic negative selection, which eliminates autoreactive clones. Consequently, mutated neoantigens—peptides derived from tumor-specific somatic mutations—are more immunogenic and represent superior targets for CTL-mediated tumor clearance [136,137]. In glioblastoma (GBM), neoantigen discovery has become an essential avenue for immunotherapy due to the disease’s genetic heterogeneity and limited mutational burden [138]. A 7 amino acid peptide sequence was able to target GBM stem cells by binding to the Cadherin 2 surface protein on GBM stem cells. Suppressive targeting of these stem cells using this peptide could provide a new avenue for GBM treatment, although its application is still experimental [139]. However, successful peptide vaccines are confined to specific HLA haplotypes, and the tumor can rapidly develop treatment resistance by applying immunoediting or by downregulating the expression of such peptide antigens [48,140]. High-throughput peptidomic and multi-omics approaches have identified approximately 30 tumor-specific antigens that are selectively expressed in GBM tissues but absent in normal cells. These antigens are often associated with oncogenic processes, such as abnormal cell signaling, proliferation, and migration. Recent studies have demonstrated that, on average, each GBM tumor harbors approximately 200 non-synonymous mutations, although there is limited overlap in mutational profiles among patients. Consequently, few neoantigens are shared between individuals, posing a significant challenge for the development of universal vaccine targets. HLA genotyping has revealed broad variability among GBM patients, with common alleles including HLA-A30:01, A11:01, B13:02, and C06:02. These alleles influence the diversity and binding preferences of MHC-presented peptides. Interestingly, while the peripheral blood harbors a diverse TCR and BCR repertoire, the repertoire within the GBM tumor microenvironment appears more restricted, dominated by NK and Th1-like immune cell populations [141].

The efficacy of peptide-based vaccines depends on effective delivery of epitopes to dendritic cells (DCs) and their subsequent presentation via MHC molecules. Synthetic long peptides (SLPs) derived from TAAs can be processed by DCs and presented to both CD4^+^ and CD8^+^ T cells. Such vaccines can induce robust, multifunctional T cell responses while minimizing the need to predefine specific tumor antigens for each patient. MHC tetramers and peptide—MHC multimers have been instrumental in identifying and quantifying antigen-specific T cells in both research and clinical settings. These tools allow precise characterization of T cell responses, enabling validation of epitope immunogenicity and tracking of clonal expansion in vivo. Importantly, HLA-A2, A24, B35, and B51 allomorphs have been shown to present a broad and diverse repertoire of tumor-derived peptides, enhancing their utility in population-wide vaccine strategies [142].

Autologous tumor cells, particularly when lysed or irradiated, serve as comprehensive sources of tumor antigens for personalized vaccine development. Unlike single-epitope vaccines, whole-tumor preparations present a broader spectrum of TAAs and neoantigens, reducing the risk of immune escape due to antigen loss. Although GBM is considered a “cold” tumor with limited neoantigen burden and poor immune infiltration, vaccination has been shown to shift the tumor microenvironment toward an inflamed, “hot” phenotype [143]. This transformation facilitates T cell infiltration and enhances the likelihood of tumor control. A subset of peptides identified in GBM have dual utility: they not only target tumor-specific receptors but also demonstrate high permeability across the blood–brain barrier (BBB), offering enhanced therapeutic delivery [144].

### 2.6. The Blood–Brain Barrier in Glioblastoma

The blood–brain barrier (BBB) is a highly specialized and selectively permeable structure that tightly regulates the exchange of molecules and cells between the systemic circulation and the central nervous system (CNS). Under physiological conditions, the BBB poses a significant obstacle to immune cell infiltration and therapeutic delivery to the brain. However, in glioblastoma (GBM), the BBB becomes structurally and functionally compromised, altering its role from a protective barrier to a permissive environment for tumor progression and therapeutic access [145].

GBM tumors induce angiogenesis and vascular remodeling, resulting in leaky, abnormal vasculature with reduced tight junction integrity. This pathological neovasculature increases permeability and disrupts normal endothelial cell function, leading to elevated interstitial fluid pressure, hypoxia, and edema. Tumor-derived factors—such as vascular endothelial growth factor (VEGF) and matrix metalloproteinases (MMPs)—degrade basement membranes and tight junction proteins, contributing to the breakdown of the BBB [146]. These structural changes facilitate local immune suppression by allowing uncontrolled infiltration of myeloid-derived cells, altering antigen presentation, and limiting the effective trafficking of activated effector T cells into the tumor parenchyma.

Despite its compromised integrity, the glioblastoma-associated BBB expresses several transport-related surface receptors that can be exploited for therapeutic purposes. These include fatty-acid binding protein 3 (FABP3/MDGI); transferrin receptor (TfR); insulin receptor; glucose transporter 1 (GLUT-1); large neutral amino acid transporter 1 (LAT-1); and low-density lipoprotein receptor-related protein 1 (LRP-1) [147]. Peptides presented by tumor-derived antigen-presenting cells or dendritic cells (DCs) following cell-based vaccination can engage CTLs trained to recognize and target tumor cells expressing these receptors, thereby promoting antigen-specific infiltration and cytotoxicity within the CNS.

The BBB presents a significant barrier to the delivery of immunotherapeutic agents, limiting their effectiveness in reaching tumor sites [148,149]. This barrier not only restricts drug transport but also alters the immune response within the central nervous system, further complicating treatment strategies [82]. While the compromised BBB in GBM is associated with tumor aggressiveness, it also presents a therapeutic opportunity. The increased permeability facilitates the entry of systemically administered or intradermally applied vaccine components, including antigen-loaded dendritic cells and cytotoxic T cells, into the CNS.

By using low-intensity pulsed ultrasound exposure in the presence of so-called microbubbles after tumor resection, the induced oscillation of these bubbles applies stress to cell walls, enabling a temporary breakdown of the BBB and allowing the vaccine to enter the extensive tumor-associated microvasculature inside the brain. This allows local and high-dosage application of vaccines or drugs [150]. Furthermore, the localized inflammation and immune cell recruitment induced by the tumor and the vaccine can help overcome the immunologically cold nature of the GBM microenvironment. Collectively, these factors support the rationale for intradermal or systemic administration of cell-based vaccines, which can leverage both the disrupted BBB and local immune activation to achieve effective anti-tumor responses within the brain.

## 3. Rationale for Cancer Transplant Immune Recognition Therapy

### 3.1. Immunological Basis

Cell-based (transplant) vaccines for GBM are therapies where autologous immune cells (usually dendritic cells) are harvested, loaded ex vivo with tumor antigens, and reinfused back into the patient to “transplant” an immune response back into the patient. Alternatively, tumor cells are obtained by resection of the tumor, tumor cells are isolated and mixed with lysates of these tumor cells, and (together with immune stimulants) the transplant is infused back into the patient where such DCs are primed in vivo. Under both circumstances, the transplant-induced immune activation of DCs induces an immune response able to attack and kill the tumor in vivo. Several clinical trials for GBM are published and comprise (cell-based) vaccines, immune checkpoint inhibitors, chimeric antigen receptor T-cell (CAR-T) therapy, and oncolytic viral therapy. However, although many immunotherapeutic agents and treatment suggestions with survival benefits are described in the literature, relatively few phase III and large-scale randomized controlled trial are published [15,151].

Autologous mono-donor tumor lysate-loaded dendritic cell vaccination (DCVax-L) and the multi-donor combined syngeneic and allogeneic tumor cells plus lysate-based vaccine Sitoiganap represent distinct cell-based vaccination strategies for glioblastoma that differ in product, adjuvanticity, and evidentiary strength (Table 1). The hallmark of these vaccines is to induce a strong and long-lasting priming of CD8^+^ and CD4^+^ T-cells specific to the individual GBM patient. Other cell-based vaccines comprise the use of heat-shock protein (gp96/HSPPC-96) complexes, as these complexes chaperone multiple tumor peptides to DCs, acting as an endogenous adjuvant [152]. In addition, antigen-focused DC vaccines include CMV pp65 RNA-pulsed DCs due to the frequent CMV antigen expression reported in GBM [153]. The hsp-based vaccine did induce immune activation and has an acceptable safety, but larger confirmatory efficacy trials are still needed [152]. The CMV pp65 DC vaccine showed strong immunogenicity and clinical long-term survivors in small cohorts, and further refinements in delivery and antigen targeting are underway [154]. Tumor cell-based therapies can stimulate the immune system by presenting tumor antigens, which may activate CD4 T cells and microglia, leading to enhanced anti-tumor activity [155,156,157]. DC vaccination combined with gene therapy (e.g., Ad-Flt3L) have shown promise in increasing the efficacy of immune responses by modifying the tumor microenvironment. The application of cell-based immunotherapies for GBM faces several significant challenges and limitations. These obstacles stem from the unique characteristics of GBM, including its immunosuppressive environment and the complexities of the central nervous system.

Working mechanism DCVax-L. At surgery, tumor cells are resected and processed into lysates containing a broad variety of tumor-associated antigens (TAAs) and neoantigens, including the known GBM antigens (e.g., EGFRvIII, IDH-related neoepitopes in some patients) plus many tumor antigens not yet predefined. After surgery, patients will undergo leukapheresis, from which monocytes are isolated and subsequently differentiated ex vivo into immature DCs using GM-CSF + IL-4. Immature DCs are pulsed with whole tumor lysate, including phagocytosis, processing and loading tumor peptides into the MHC I and II molecules to present and activate CD4^+^ and CD8^+^ T-cells by cross-presentation. A mix of cytokines is added (e.g., IL-1β, TNF-α, IL-6, PGE2 or similar) to help mature the DCs, as evidenced by upregulating the expression of MHC, CD40, CD80, CD86 and CCR7 [159]. About 10^7^ of these antigen-loaded DCs are injected intradermally back into the patient where they migrate to regional lymph nodes, guided by chemokine signals, and where they present tumor antigens to both CD8^+^ cytotoxic T cells and by cross-presentation to CD4^+^ helper T cells (see Section 2.2). These primed T cells differentiate into tumor-specific cytotoxic T cells (CTL) that are capable of infiltrating the brain tumor environment and lyse tumor cells by the perforin/granzyme and Fas–FasLigand pathways [160]. The CD4^+^ T helper cells secrete cytokines (IL-2, IFN-γ) and amplify the expansion of CTL cells while also promoting antibody production by B cells. Both T cell populations also give rise to the formation of memory cells that sustain long-term immune surveillance against recurrence of the tumor. When using lysates of tumor cells, more antigens are released and loaded into the DCs and thereby broadening the repertoire of responding T cells, which is called epitope spreading, as over 300 epitopes of 9 amino acids are loaded into MHC class I molecules, while over 200 epitopes of about 16 amino acids are loaded into MHC class II molecules. This is important to arm T cells against the highly heterogeneous GBM tumor. However, GBM remains strongly immunosuppressive (PD-L1, myeloid cells, steroids, etc.), so responses are often incomplete, which is why combinations (checkpoint blockade, oncolytic viruses, RT, TMZ timing) are heavily discussed. The DCVax-L-induced T-cell infiltration in the brain can shift the tumor microenvironment from a “cold” immunosuppressive (dominated by Tregs, M2 macrophages, and PD-L1 expression) toward a more “hot” pro-inflammatory, immune-active state, thereby enhancing antigen spreading characterized by memory immune responses against additional tumor antigens released during tumor lysis.

**Figure 2:**
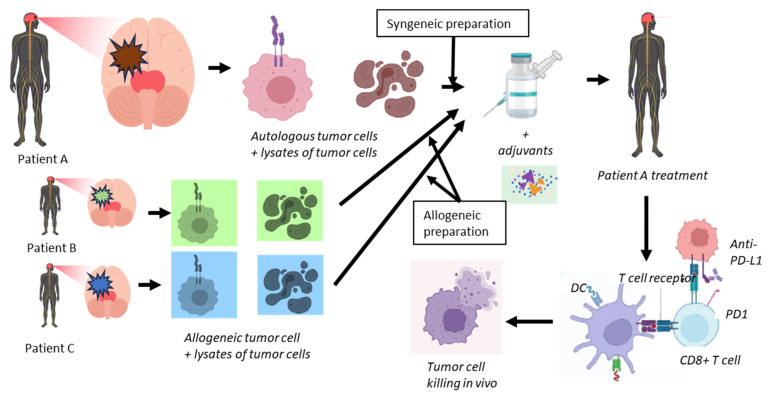
Outline of the treatment using Cancer Transplant Immune Recognition Therapy of newly diagnosed but also recurrent GBM patients. From a patient (A), tumor will be resected and prepared by isolating intact individual tumor cells (syngeneic preparation). After irradiation and haptenization of these cells, they will be mixed with lysates of another aliquot of these isolated tumor cells. This preparation will be mixed with similar preparations obtained from other patients (B and C) (allogeneic preparation). For clarity, in this figure, only two allogeneic preparations are shown, while in the Sitoiganap treatment scheme, three allogeneic preparations are used. This mix of cells and lysates will be complemented with GM-CSF, a low dose of cyclophosphamide and irradiated and the addition of anti-PD-L1 specific monoclonal antibodies. Patient A will be treated with alternating preparations of syngeneic and allogeneic cell plus lysate preparations to ensure optimal in vivo expansion of tumor-specific CD8^+^ T-cells, depending on the applied protocol. Upon infusion into patient A, the dendritic cells DCs (by cross-presentation) will be loaded with tumor-associated and tumor-specific antigens which will be presented to antigen-specific CD4+ helper T cells and cytotoxic CD8^+^ T cells. The latter cells will be provided by the CD4+ T cells with T cell, which help to allow them to differentiate into killer cells that target the residual tumor cells in vivo.

Working mechanism Sitoiganap. To address the immune evasion in GBM, cell-based therapeutic tumor vaccines were developed [24,25,41,52,53,81,82]. In order to boost the induction of a strong cytotoxic T-cell response, tumor cells derived from the combination of autologous and allogeneic tumors provide a collection of tumor-specific peptide T-cell antigens. The vaccine is designed to induce a potent oligoclonal cytotoxic T cell response that not only recognizes tumor-specific antigens but also mounts an alloimmune response due to HLA mismatch (Figure 2). By using freshly resected tumor tissues from the patient and from unrelated GBM donors, a broad-spectrum antigen presentation with relevant epitopes is induced. Rather than selecting specific tumor epitopes, whole tumor lysates represent a repertoire of tumor antigens to a patient’s immune system for the subsequent selection and expansion of pre-existing multiple tumor-specific CD8^+^ T-lymphocyte clones [53]. Activation of these alloreactive CD8^+^ T cells occurs at much higher precursor frequencies than tumor antigen-specific T cells in combination with breaking immune tolerance via mismatched allo-antigens, which results in a robust bystander activation of dendritic cells and inflammatory cytokine production, converting the tumor microenvironment into a more immunostimulatory setting [161]. In addition, autologous tumor components ensure that the activated immune response is directed toward the patient’s unique tumor antigen repertoire, enhancing specificity and reducing the risk of off-target effects. This strategy, termed Cancer Transplant Immune Recognition Therapy (CTIRT), reduces the risk of immune escape, and bypasses the need to predefine immunogenic peptides [48,49,52,53].

CD8^+^ T cells reactive to tumor-associated or allogeneic MHC-peptide complexes exist at frequencies of approximately 1 in 10^3^–10^5^ within the naïve repertoire. Upon vaccination, these cells undergo rapid in vivo expansion, reaching levels of 10^9^–10^10^ total tumor-specific cytotoxic T lymphocytes (CTLs), a population size sufficient to mediate significant tumor burden reduction [162,163]. The vaccine’s efficacy does not depend on predefined antigens or specific T cell receptor (TCR) profiles. Instead, it leverages antigenic diversity and HLA mismatches to stimulate a broad cytotoxic response. This eliminates the need for individualized TCR engineering or MHC-peptide matching, which can be both time-consuming and technically complex. A minimum of 1 cm^3^ of tumor tissue (approximately 1 g wet weight, containing ~1 × 10^9^ tumor cells) is required from the patient to generate sufficient autologous vaccine material [164]. This quantity allows for the preparation of multiple vaccine doses, supporting both priming and booster immunizations throughout the treatment protocol. The allogeneic vaccine components are derived from tumor cell banks created from consenting GBM donors [165].

### 3.2. Clinical Reactivity

Autologous dendritic cell vaccines have been reported to be safe and well-tolerated, with minimal adverse effects such as fever and rash [23,165]. Also, treated patients exhibited increased tumor-associated antigen-specific CD8 T-cell immune responses post-vaccination, indicating an active immune engagement [166,167,168,169,170,171,172,173]. DCVax-L treatment in a phase III trial in both newly diagnosed and recurrent GBM patients demonstrated an improved clinical survival advantage compared with matched controls, although progression-free survival did not show improvement [23]. Increased tumor-infiltrating lymphocytes (TILs) and circulating IFN-γ and IL-2, as well as increased T cell memory populations, were found post-vaccination in survivors. These results suggest that DCVax-L can provide a complementary immunotherapy protocol following surgery and chemoradiation.

The clinical response of the autologous and allogeneic tumor-based Sitoiganap vaccine has shown results with respect to median overall survival and progression-free survival in newly diagnosed GBM patients [24,25,40,174,175]. Clinical and preclinical studies have demonstrated that the dual use of syngeneic and allogeneic tumor cell-based Sitoiganap vaccines elicits infiltration of tumor-reactive lymphocytes, including neoantigen-specific CD8^+^ T cells [176]. Moreover, the incorporation of multiple donor tumor sources increases the diversity of presented epitopes and the breadth of the CTL response, allowing effective tumor clearance in the context of GBM’s genetic heterogeneity. The degree of HLA mismatch, particularly involving class I HLA-B and class II HLA-DRB1 loci, has been shown to correlate with the strength of the allogeneic response. Despite these advances, the immunosuppressive nature of the GBM tumor microenvironment and the IL-10- and TGF-β-based immune evasion strategies presents ongoing challenges, which must be overcome for therapeutic efficacy.

These donor cells are irradiated to prevent proliferation and reduce the risk of graft-versus-host disease while retaining antigenicity. During irradiation, donor tumor cells may acquire neoantigens due to genetic drift or stress-induced mutations, and this increased immunogenicity correlates with the degree of HLA mismatch, particularly involving class I HLA-B and class II HLA-DRB1 loci [177,178]. This epitope spreading helps to deal with the appearance of antigen-loss variants and counter tumor heterogeneity. The application of haptenization of the tumor cells involves covalently attaching a small chemical group, called a hapten (DNP, TNBS), to proteins on or in tumor cells. This procedure alters the (generally) poor tumor immunogenicity into highly immunogenic material and creates neoepitopes that can be presented by DC to both CD4^+^ and CD8^+^ T cells and thereby break immune tolerance [179]. The initial CTL response can destroy the haptenated tumor cells, resulting in tumor antigen release and subsequent DC presentation of these unchanged, native tumor epitopes, expanding T-cell specificity beyond the hapten targets.

Allogeneic Tumor Lysate Vaccines, combined with standard therapies like temozolomide, have shown clinical efficacy associated with prolonged survival [158]. However, the clinical results are variable and often modest, despite a good safety profile with no reported dose-limiting toxicities [132]. The multiple rounds of vaccine treatment (Figure 2) enhance the immune response specificity in GBM patients by presenting unique tumor-associated antigenic peptides, thereby minimizing potential immune escape, and stimulating targeted oligoclonal immune pathways that recognize and attack the patient’s own tumor effectively [36,37,40]. Recently, the proposed integration of immunotherapeutic strategies, including checkpoint inhibitors and DC vaccines, aims to reshape the GBM microenvironment, potentially improving patient outcomes [84]. Ongoing research is focused on understanding the cellular interactions within the GBM microenvironment to develop more effective immunotherapies [180,181]. Conversely, while allogeneic tumor cell-based immunotherapy shows potential, challenges remain due to the complex and adaptive nature of the GBM microenvironment, which may still hinder the effectiveness of these treatments.

Current challenges of allogeneic cell-based immunotherapy for glioblastoma include the immune-protective mechanisms in the CNS, the immunosuppressive tumor microenvironment, low tumor mutation burden limiting neoantigen presentation, and the heterogeneity of GBM cells, which contribute to treatment resistance, ineffective immune activation, and poor clinical outcomes compared to other solid tumors [9,182,183,184]. In addition, the blood–brain barrier’s restrictive nature and the rapid adaptation of tumors through antigen escape complicate effective treatment and resistance management, necessitating broader antigen targeting and combination regimens for future efficacy [185,186]. Current challenges of allogeneic cell-based immunotherapy for glioblastoma also include T-cell dysfunction in patients, complex immune dysfunction, and constraints in drug delivery to the central nervous system, which hinder effective treatment outcomes and necessitate innovative combinational regimens and novel targets [149,187].

### 3.3. Other Vaccine Compounds

To further improve the immune response programming activity of such a cell-based vaccine intended for Cancer Transplant Immune Recognition Therapy, Granulocyte–Macrophage Colony-Stimulating Factor (GM-CSF) is used as immunological adjuvant, which is known to facilitate and increase antigen presentation in distinct types of cancers. In addition, a low dose of cyclophosphamide precedes the treatment to deplete immunosuppressive regulatory T-cells [188].

GBM uses several signaling transduction pathways, including the path involving PD-1, to negatively regulate cytotoxic T-cells and Tregs allowing tumor proliferation and invasion of neighboring tissues. PD-1 is expressed in T-cells, B cells, and NK cells, thus correlating with adaptive and innate immune responses, and this contributes to the suppressive tumor environment [15,22,128]. Overexpression of PD-L1 was reported in 88% of newly diagnosed and 72.2% of recurrent GBM specimens [189]. Blocking PD-1, or its ligand (PDL1), inhibits the interaction of PD-L1 and PD-1 receptor to mitigate the suppression of CTLs, but also induces anti-tumor immune responses [190]. Therefore, cell-based vaccines combine tumors cells and/or their lysates with GM-CSF, and anti-PD-1 monoclonal antibodies, which results in significant improvements in patient outcomes [24,25].

Isolated tumor cells are haptenized with 1-fluoro 2,4-dinitrofluorobenzene (DNFB) to improve immunogenicity when re-introduced. The frequency of hapten-specific T cells is about 1:106 cells, which would equal 10^3^ responding cells in 10^9^ total naïve T cells [89,191]. It has been established that the modification of autologous cancer cells with the hapten, dinitrophenyl (DNP), induces a unique reaction: the development of inflammation in metastatic masses. The inflammation is mediated by IFN-γ-producing CD4^+^ Th1 and CD8^+^ CTL lymphocytes, some of which represent the expansion of novel clones. Following DNP-vaccine treatment, almost all patients develop delayed-type hypersensitivity (DTH) to autologous, DNP-modified tumor cells; approximately half also exhibit DTH to autologous, unmodified tumor cells. The toxicity of DNP-modified cells in a tumor vaccine is generally mild, consisting mainly of papules or pustules at the injection sites.

In addition, these tumor cells in the vaccine are irradiated to prevent renewed tumor growth when transferred back into the patient, and even a possible graft-versus-host response as residual immune cells present in the cellular part of the treatment can recognize and attack the recipient tissue, since this is recognized as non-self.

### 3.4. Potential Biomarkers for GBM Immunotherapy

Identifying glioblastoma (GBM) patients who would benefit most from allogeneic tumor cell-based immunotherapy involves understanding various biomarkers and predictive factors [192,193,194]. These biomarkers can help stratify patients and improve the efficacy of immunotherapy. Several studies have explored different approaches to identify these biomarkers, including genetic, molecular, and computational methodologies. Neoantigens, which are tumor-specific antigens arising from somatic mutations, have been identified as potential predictive biomarkers for immunotherapy response in GBM. They can serve as targets for personalized immunotherapy approaches [195]. An artificial neural network model identified a 20-gene panel that predicts immunotherapy response and survival benefits in GBM patients treated with immune checkpoint inhibitors (ICIs). This panel demonstrated high predictive accuracy, with an AUC of 0.97, indicating its potential as a biomarker for selecting patients likely to benefit from ICIs [102]. A novel computational framework identified Tumor-Infiltrating Immune Cell-Associated lncRNAs (TIIClnc) signatures that correlate with immune cell infiltration levels and predict immunotherapy response. These signatures showed superior performance in predicting survival outcomes compared to existing signatures [186].

However, the heterogeneity of GBM poses a challenge in identifying universal biomarkers. Currently, apart from microsatellite instability (MSI), only a few biomarkers can be used for patient stratification in GBM immunotherapy [196,197]. By combining multiple biomarkers, such as genetic, molecular, and immune cell-associated factors, the precision of patient selection for immunotherapy may be enhanced [196,197,198,199]. While these biomarkers show promise, the complexity and heterogeneity of GBM necessitate further research to validate and integrate these findings into clinical practice [200,201]. The development of comprehensive biomarker panels and computational models could lead to more personalized and effective immunotherapy strategies for GBM patients.

## 4. Conclusions

**Figure 3:**
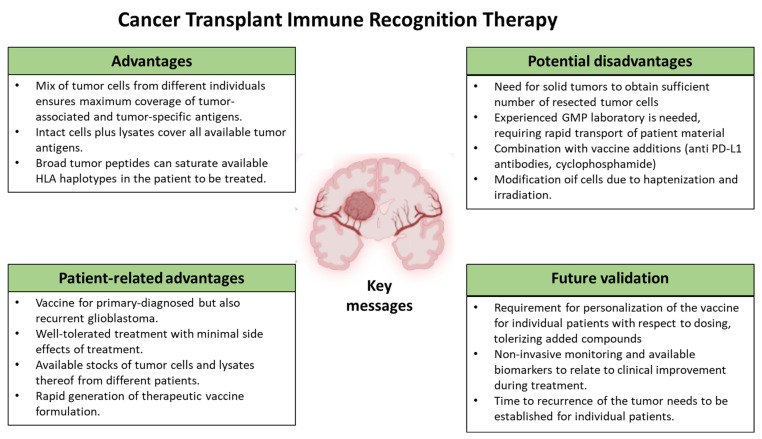
Key messages of the Cancer Transplant Immune Recognition Therapy as outlined in this review.

Despite various promising results from studies on GBM immunotherapies, the variability in these results and the multitude of therapeutic targets make it challenging to translate these results into clinical success for GBM patients (see Figure 3 for the key messages of this Cancer Transplant Immune Recognition Therapy). Currently, the ongoing studies are using combination approaches and enhancing existing immunotherapeutic strategies, thereby offering hope for a revolutionary breakthrough in GBM cancer care that is urgently needed. Besides the infusion of ex vivo DC primed with autologous tumor lysates (DCvax-L), the infusion of combinations of cells and lysates of autologous and allogeneic glioma cells (Sitoiganap) can also be used to stimulate a robust, polyclonal CD8^+^ T cell response capable of mediating tumor regression. Both strategies are promising as adjuvant therapy after post-surgical resection or as a standalone treatment in non-resectable cases. By combining tumor-specific and alloantigen-driven immune activation, this approach leverages a novel immunotherapeutic strategy that bypasses the need for predefined tumor-associated antigens, instead capitalizing on the immunogenic potential of HLA mismatches to break tolerance and overcome the immunosuppressive glioblastoma microenvironment. The inclusion of immunostimulatory adjuvants, such as GM-CSF and anti-PD-L1 antibodies, and regulatory T cell depletion via low-dose cyclophosphamide, further enhances immune activation, while irradiation ensures vaccine safety by preventing tumor cell replication. Clinical studies to date support a favorable safety profile and meaningful survival benefit, underscoring its potential role in future standard-of-care protocols for recurrent and newly diagnosed glioblastoma. This strategy represents a paradigm shift in the treatment of high-grade gliomas, particularly in patients who are refractory to conventional therapies.

## Figures and Tables

**Table 1:** Comparison of two major cell-based vaccine strategies for GBM patients.

	DCvax-L	Sitoiganap
Donor	Mono-donor	Multi-donor
	Monocyte-derived DC ex vivo	In vivo DC subsets
Vaccine basis	Autologous tumor lysate	Allo/auto mixture of irradiated whole tumor cells plus tumor lysates
Adjuvants		Treg inhibitor (low-dose cyclophosphamide) + GM-CSF + VEGF blockade (bevacizumab) + PD-1 blockade (pembrolizumab)
TMZ addition	+TMZ	+TMZ
Study phase	Phase III	Phase II
Newly diagnosed GBM survival	Median OS: 19.3 vs. 16.5 months	Median OS: 12 vs. 7.5 months
Recurrent GBM survival	Median OS: 13.2 vs. 7.8 months	Median OS: 19.6 vs. 7.5 months
Trials	Phase III	Phase II
References	[23,158]	[24,25,60]
